# Alpha-Tocopherol Serum Levels Are Increased in Caucasian Women with Uterine Fibroids: A Pilot Study

**DOI:** 10.1155/2018/6793726

**Published:** 2018-07-24

**Authors:** Michał Ciebiera, Jolanta Szymańska-Majchrzak, Aleksandra Sentkowska, Krzysztof Kilian, Zbigniew Rogulski, Grażyna Nowicka, Grzegorz Jakiel, Piotr Tomaszewski, Marta Włodarczyk

**Affiliations:** ^1^First Department of Obstetrics and Gynecology, Centre of Postgraduate Medical Education, Ul. Czerniakowska 231, 00-416 Warsaw, Poland; ^2^Department of Biochemistry, Medical University of Warsaw, Ul. Banacha 1b, 02-093 Warsaw, Poland; ^3^Heavy Ion Laboratory, University of Warsaw, Ul. Pasteura 5a, 02-093 Warsaw, Poland; ^4^Radiochemistry for Medicine and Industry, Biological and Chemical Research Centre, University of Warsaw, Ul. Żwirki i Wigury 101, 02-089 Warsaw, Poland; ^5^Department of Biochemistry and Pharmacogenomics, Medical University of Warsaw, Ul. Banacha 1b, 02-093 Warsaw, Poland; ^6^Department of Biochemistry, Second Faculty of Medicine, Medical University of Warsaw, Warsaw Poland, Ul. Banacha 1, 02-097 Warsaw, Poland; ^7^University of Ecology and Management, Olszewska 12, 00-792 Warsaw, Poland

## Abstract

Uterine fibroids (UFs) are benign tumors of the reproductive tract, arising from smooth muscle cells of the uterus. Steroid hormones, estrogen, and progesterone are considered to be the most important links in the pathophysiology of UFs. Alpha-tocopherol (AT) is the most active form of vitamin E. What is important as far as UFs are concerned is that ATs contain structural determinants, which makes them possible ligands for estrogen receptors (ERs). We present a retrospective cohort study performed in a university teaching hospital. We included a total of 162 patients divided into 2 groups: with UFs and controls. The effects of age, body mass index (BMI), positive medical history, parity, and AT serum concentrations on the risk for the development of UFs were investigated. Mean AT serum concentrations were 11.66 ± 4.97 *μ*g/ml and 7.83 ± 3.13 *μ*g/ml (medians 10.56 *μ*g/ml and 7.42 *μ*g/ml) in patients with UFs confirmed on ultrasound and controls, respectively. The presented difference was statistically significant. Higher BMI, positive family history, and low parity were found to be major risk factors for UFs. In our study, we confirmed that elevated serum AT concentration might be an important risk factor for UFs in Caucasian women. Further research in this area is necessary.

## 1. Introduction

Uterine fibroids (UFs) are benign tumors of the reproductive tract, arising from smooth muscle cells of the uterus. As many as 80% of women may develop UFs, which is associated with several risk factors, mainly age and ethnicity [1, 2]. Depending on their localization and size, UFs may cause different clinical symptoms, including abnormal uterine bleeding, iron-deficiency anemia, pain, constipation, bloating, polyuria, dyspareunia, infertility, and many others [1–4]. Clinical symptoms of sufficient intensity may impair normal functioning in approximately one-fourth to one-third of the affected patients [1, 5]. UFs are a major public health concern [2] and constitute a significant burden on healthcare budgets of most countries around the world, regardless of the level of their development. Furthermore, UFs are one of the major reasons for surgery in women [2, 6–8]. What is more, they can be recurrent and often require repeated surgical interventions [8]. Also, other UF-associated costs, including pharmacological treatment, hygiene accessories, imaging diagnostics, and absenteeism at work, ought to be mentioned as well [7, 9].

Despite extensive research, the etiology of UF development and growth remains to be fully elucidated [2, 3]. Steroid hormones, estrogen, and progesterone are considered to be the most important links in the pathophysiology of UFs [3, 4, 10, 11], whereas progesterone is currently believed to be the key factor in the processes occurring within the UFs [3, 4, 12]. The risk factors for the development of UFs are better known than their etiology [1] and include elevated BMI values [1, 13, 14], positive family history [1, 4, 14], genetics [15, 16], black race [17, 18] hypovitaminosis D [14, 19, 20], soybean and food additives consumption [1], and elevated serum TGF-*β*3 (transforming growth factor beta 3) levels [14, 19, 21]. Obviously, the list of the abovementioned risk factors is not complete and further research in this area is necessary. Identification of women at higher risk for UFs and UF-derived symptoms remains the ultimate goal of the research.

Transformation of uterine smooth muscle cells into abnormal, immortal cells, which are capable of clonal division, is the major component of all pathways leading to the formation of a fibroid tumor [3, 11, 16, 22]. According to recent studies, mutations, especially those in the MED12 gene [3, 15, 23], and oxidative stress [24, 25] are of key factors in this regard. Oxidative stress is an imbalance between a systemic manifestation of reactive oxygen species (ROS) and tissue ability to readily detoxify the reactive forms or to repair the resulting damage [26]. Stressors from the environment, like ionizing radiations, heavy metals, or xenobiotics, greatly contribute to the increase in ROS production, which leads to cell and tissue damage [26]. UFs have an impaired antioxidant system, which presents with lower activities of superoxide dismutase and catalase in comparison to normal uterine smooth muscle cells [24, 25]. In a recent study, Fletcher et al. demonstrated that enhanced oxidative stress is associated with decreased apoptosis and might change normal myometrial cells to fibroid-like cells [25].

An antioxidant is a molecule which inhibits oxidation of other molecules [27]. There are several different antioxidant systems, e.g., enzymes, such as the abovementioned catalase and superoxide dismutase, but there are also dietary antioxidants, like vitamins A, C, or E [28]. Vitamin E is a collective name for a group of different compounds which include tocopherols (with saturated isoprenoid chain) and tocotrienols (with unsaturated isoprenoid chain) [29, 30]. Vitamin E was first characterized in wheat germ oil and lettuce in 1922 [30]. Tocopherols can be found also in corn oil, soybean oil, margarine, sunflower, or safflower oils [31]. Alpha-tocopherol (AT) is the most active form of vitamin E and the second most common form of vitamin E in the diet, after *γ*-tocopherol [32]. AT is absorbed in the intestine and transported to the liver [33, 34], where it is immediately transferred to the *α*-tocopherol transfer protein (*α*-TTP) and then moved to other tissues [33, 34]. The biological activity of vitamin E is dependent upon *α*-TTP regulation. This protein recognizes AT and, as a result of this specificity, AT is protected against rapid metabolism of phase I and II enzymes [33, 34].

What is important as far as UFs are concerned is that ATs contain structural determinants, which makes them possible ligands for estrogen receptors (ERs) [30, 35]. In a study by Hashemi et al., a 12-week supplementation with vitamin E among women with implantation failure had beneficial effects on the endometrial thickness [36]. Vitamin E is also known to be beneficial for postmenopausal women as it helps to relieve symptoms like night sweats or hot flashes [26, 37]. In some cases, vitamin E remarkably improves the release of paracrine factors like epidermal growth factor (EGF) or vascular endothelial growth factor (VEGF) [38, 39].

As a result of most guidelines [40] and advertisements in the media, many women supplement vitamin E, treating it as a ‘beauty and health' panaceum. In light of the fact that most supplements contain AT, we decided to investigate whether there is a relationship between AT serum concentrations and the risk for UFs in women.

## 2. Objectives

The aims of the study were as follows: (i) to evaluate and compare serum AT concentrations in patients with UFs and controls; (ii) to evaluate the impact of serum AT concentrations, age, BMI, positive medical history, and parity on the risk of developing UFs.

## 3. Material and Methods

### 3.1. Groups

A total of 162 Caucasian women, recruited among the patients of the First Department of Obstetrics and Gynecology, Centre of Postgraduate Medical Education, Warsaw, Poland, were included in the study (from September 2014 to May 2015). The subjects were subdivided into 2 groups: women admitted to hospital for surgery due to UF-derived clinical symptoms, e.g., abnormal uterine bleeding, anemia, abdominal and pelvic pain, gastrointestinal disorders, and infertility (UF-positive patients), and controls, recruited among the outpatient clinic patients during control visits and including women without identified UF (UF-negative). All subjects had gynecological examination and a gynecological ultrasound scan, completed a questionnaire, and had selected biochemical parameters analysis. Informed written consent was obtained from all participants. Local Ethics Committee approved the study. The inclusion criteria for UF-positive patients were as follows: age > 18 years, at least 1 UF (min. 10mm in diameter) on transvaginal or transabdominal ultrasound exam, and eligibility for surgical removal of the UF or surgical removal of the uterus due to severe clinical symptoms. The control group inclusion criteria were as follows: age > 18 years; no UFs on transabdominal or transvaginal ultrasound exam. The exclusion criteria for both groups were as follows: vitamin E supplementation during the last 6 months, eating disorders, restrictive diets, menopause, pregnancy, active neoplastic disease, or history of malignancy.

Gynecological ultrasound scans (transvaginal or/and transabdominal) were conducted by a certified physician using General Electric Voluson E8 BT 13 with transvaginal I-C 5-9 D probe and abdominal RAB 4-8 D probe. A UF was defined as a hypoechogenic and heterogenous symmetric area with distinct margins.

### 3.2. Determination of Serum *α*-Tocopherol

Blood samples were collected for biochemical analysis in fasting patients. In UF-positive patients, the blood samples were taken one day before surgery. Serum AT was measured using high-performance liquid chromatography (HPLC) method.

#### 3.2.1. Instrumentation

Chromatographic analysis was carried out using the Shimadzu LC system consisted of binary pumps LC20-AD, degasser DGU-20A5, column oven CTO-20AC, autosampler SIL-20AC, and 8030 Mass spectrometer (Shimadzu, Japan). A MS system was equipped with electrospray ionization source (ESI) operated in negative or in positive-ion mode according to species being determined. Evaporation of solvents during the preparation the samples was performed using Eppendorf® centrifugal vacuum concentrator. The chemicals were supplied by Sigma-Aldrich.

#### 3.2.2. Samples Preparation

Blood samples (5 ml) were collected in serum tubes and centrifuged at 3000 rpm for 5 min; serum samples were put in 0.5-ml microtubes and stored at -20°C until usage. Concentration of *α*-tocopherol in serum samples was determined by the modified procedure described by Cheng et al. [41]

Briefly, thawed plasma samples (0.1 mL) were added to 0.1 mL of *α*-tocopheryl acetate (25 *μ*g/mL), which was an internal standard, and 0.1 ml of 1% pyrogallol in ice-cold ethanol. Samples were vortexed for 1 min and then 1.5 mL of n-hexane was added to obtained solution, followed by 4 min of vortexing. After centrifugation at 1100 rpm for 5 min the upper layer containing hexane was collected and evaporated at 45°C using concentrator. Obtained residue was dissolved in 0.1 mL of methanol: chloroform (2:1, v/v) and analyzed by reversed phase chromatography LCMS. 10 uL of the sample was injected to the chromatograph, equipped with Kinetex C18 (150 x 3.00 mm, 2.6um) column. The isocratic separation was conducted in 95:5 v/v methanol:8mmol/L formic acid (pH 2.8) with 0.4 mL/min flow. Column temperature was 30°C. ESI conditions were as follows: capillary voltage 4.5 kV, DL temperature and Heat Block temperature 230°C, the source gas flow 2 L/min, and drying gas flow 15 L/min. Detection of positive ions in MRM mode was as follows: AT (precursor m/z 430.60, products (m/z, collision energy eV) 165.00, -34; 163.95, -38; 293.95,-30); tocopherol acetate (internal standard) (precursor m/z 473.25, and products (m/z, collision energy eV) 207.20, -21; 165.20, -48; 69.10,-43). The attempt in negative ions mode resulted in lower sensitivity and higher limit of detection.

### 3.3. Statistical Analysis

All analyses were performed using Statistica software (version 12). Data are presented as median with interquartile range (IQR) and mean (±standard deviation) for continuous variables and number of subjects (%) for categorical variables. Normality of data distribution was assessed with the Shapiro-Wilk test. Comparisons between control and UF groups were made using the Mann-Whitney test, and the relationship between variables was tested with Spearman's rank correlation analysis. Categorical variables were tested using the *χ*2 test.

Studied subjects were divided into quartiles based on the data of AT concentration pooled across the cases and control groups. The categories of covariates included in the logistic regression models were as follows: age, obesity (yes or no), delivery (at last one or none), family history of UF (yes or no), and quartiles of AT (<7.26 *μ*g/ml, 7.26 to 9.09 *μ*g/ml, 9.10 to 11.92 *μ*g/ml, >11.92 *μ*g/ml). The odds ratio (OR) and 95% confidence interval (CI) of UF for participants in the second, third, and fourth quartile were calculated relative to the first quartile. Crude and age-adjusted analysis were applied.* p*-values for trend were performed by assigning the median value of each quartile and then modelling this as a continuous variable in a separate regression model.* p*-value <0.05 was considered as statistically significant.

## 4. Results

**Figure 1:**
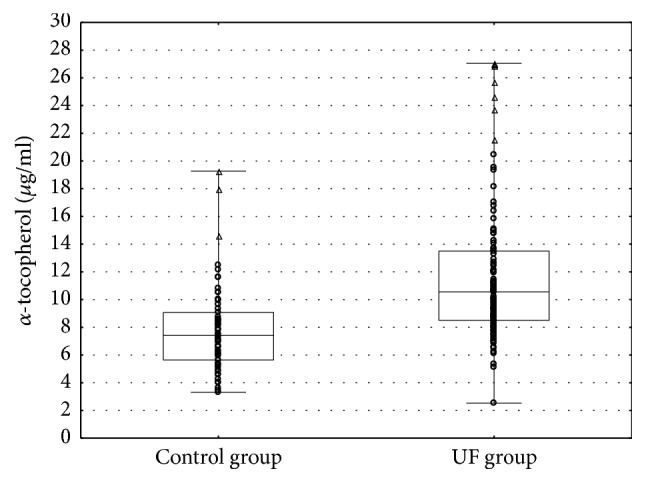
The level of *α*-tocopherol (*μ*g/ml) of the control and UF group. Boxplots show median, upper, and lower quartiles and minimum and maximum data.

Characteristics of the study subjects are presented in Table 1. Women with UFs had higher mean weight and BMI values. Mean serum AT concentration in all studied subjects was 10.19±4.73 *μ*g/ml (min: 2.53 *μ*g/ml; max: 27.05 *μ*g/ml). As shown Figure 1, women with UFs had significantly higher mean AT concentrations as compared to women without UFs (11.66 *μ*g/ml versus 7.83 *μ*g/ml;* p* < 0.001).

**Figure 2:**
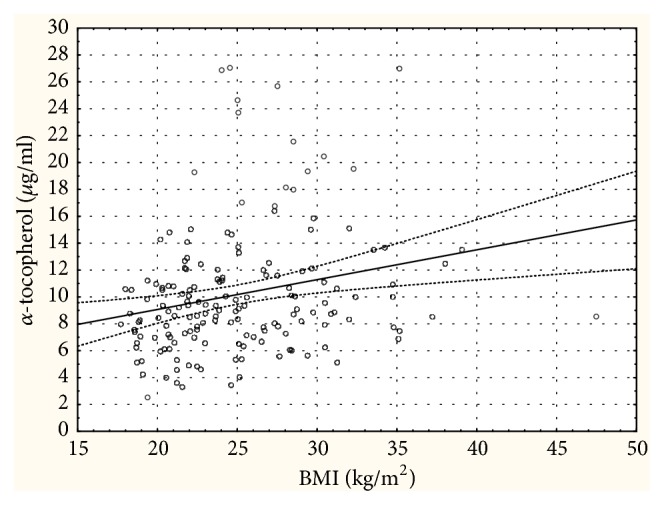
Relationship between BMI and *α*-tocopherol in all studied subjects. Dashed lines indicate 95% prediction intervals for the regression: R* *=* *0.263; p=0.001.

Figure 2 shows a weak significant correlation between AT and BMI observed in all subjects (R* *=* *0.263, p=0.001). However, in separated analysis this relationship was no longer statistically significant: R=0.211, p=0.179 in control group and R= 0.135, p=0.100 in UF group.

Table 2 presents the frequency of the studied risk factors for UF occurrence and results of crude and age-adjusted regression analysis. The family history of UFs was more frequent in the UF group than in the control subjects (42% versus 14.5%). Higher obesity rates were found among UF women as compared to the controls (21% versus 6.5%). Also, 74% of nulliparas had UFs.

Crude logistic regression analysis showed that obesity (BMI ≥30 kg/m^2^) and UFs in a family were significantly associated with an increased risk for UFs (Table 2). Whereas one or more delivery decreased the risk for UF by about 50% (OR=0.50; 95% CI: 0.25-0.96). Adjustment for age did not alter significantly the main results.

In the statistical analyses, we used quartiles of AT measurements. Women with AT in the range of 7.26-9.09 *μ*g/ml had an almost 3-fold higher odds ratio of UF compared with those with the lowest AT category (OR=2.91, 95% CI: 1.11-7.63) (Table 2). Among women in the highest quartile of AT the age-adjusted risk was more than 18-fold higher compared with women in the lowest quartile (OR=18.05, 95% CI: 4.81-67.79). AT showed significant trend of increasing risk for developing UF with increasing levels of AT, p_trend_ = 0.004 in crude analysis, and p_trend_ = 0.051 after adjustment for age.

## 5. Discussion

To the best of our knowledge, this has been the first study to show a potentially adverse effect of AT on the risk for UFs. The results are especially intriguing, because for many years vitamin E, as an antioxidant, was thought to protect against the occurrence of these tumors. There are already some studies which tested serum AT levels and the risk for UFs [42, 43]; however, they are still a rarity. In a study by Martin et al., based on the National Health and Nutrition Examination Survey, a dose-response relationship between vitamin E and UFs was observed, but after adjustment for age and race, the results were found to be statistically insignificant [42]. According to authors of the cited study, it had a limitation in a sample size which was too small to produce reliable results for specific demographic features, e.g., different races that might have been an important modifier in studied associations [42]. Wise et al. found that a greater dietary intake of fruits and vitamin A reduced the risk for UFs [43]. This was not confirmed in the case of other vitamins, but the obtained results were close to statistical significance (in the highest quintiles, higher percentages of UFs occurrence were observed) despite heterogeneous populations, where many factors could have modified AT concentrations [43]. In this study, not all participants were screened for UFs and some of the UF-positive women might have been counted as UF-negative, particularly those with asymptomatic tumors [43]. The second difference between this and our study is that the study by Wise et al. was limited only to black women [43].

As mentioned above main advantage of our work is a very homogeneous group of Caucasian women and that, despite surprising AT results, the rest of the main population risk factors coincide with those which have been confirmed earlier [2], i.e., family history [1, 14, 44], obesity [13, 14], and nulliparity [1]. The main limitation of our work is the sample size, but most of the results are of strong statistical significance.

However, the question remains what may be the reason that in this case vitamin E turned out to be a risk factor even stronger than family history (Table 2). Many compounds may have potential implications for the treatment of UFs, and the research is ongoing [45]. Some in vitro studies indicated that vitamin E may be used in the treatment of UFs [46]. However, according to many recent randomized control trials, dietary supplements (including vitamin E) neither improve health or mortality rates nor are effective in disease prevention [47–50]. Moreover, in a SELECT study, vitamin E supplementation increased the risk for prostate cancer in men [50].

As stated above, the development of UFs is determined by several factors, but steroids (estrogen and progesterone) and their receptors seem to play a major role in this process [3, 4, 12]. UFs do not appear before menarche and decrease in size after menopause. The paracrine effect of steroid hormones greatly affects the entire process of myometrial transformation [3, 22, 51]. According to recent data, the proliferative potential and UF growth are associated mostly with the progesterone and progesterone-induced growth factors [3, 12, 52–55], and the role of estrogen should not be forgotten [56]. These hormones play a major role in the entire female reproductive physiology and pathophysiology, with the additional role in other systems [56]. There are several different aberrations in estrogen-related pathways in UFs, including genomic and nongenomic ones [56, 57].

ATs have been known for their excellent antioxidant properties [29, 58]. Vitamin E acts as a peroxyl radical scavenger; it reacts with ROS and forms tocopheryl radicals [29, 58, 59]. It also has an effect on the expression of selected genes (e.g., connective tissue growth factor (CTGF) and others), which are important in wound repair and regeneration [38, 39, 60]. In a very recent study by Mancio et al., vitamin E improved skeletal muscle injury and promoted membrane repair in mouse models [61]. There are numerous studies which suggest that tocopherols can help in preventing or modulating diseases associated with oxidative stress, such as cardiovascular diseases [62] and cancers [62, 63]. There are also some laboratory studies which reported a beneficial effect of vitamin E on UF cells [46]. In their study Young et al. grow the UF cells in the presence of different agents (vitamin E succinate, thiazolidinedione, and vitamin C) [46]. In this study pioglitazone and rosiglitazone inhibited proliferation, while vitamin E succinate reduced UF cell number; authors concluded that vitamin E might induce cell death in UF cells cultures [46]. However, according to more recent data, experts failed to establish any preventive effects of vitamin E on tumors [64].

It is often forgotten that ATs also have estrogen-like properties [30, 65]. In case of UFs, these properties may be the key component of the fact that some of their concentrations may be a risk factor for the occurrence and growth of UFs. ATs contain structural elements which may be potential ligands for ER [30, 35]. According to Anstead et al., these are cyclic structure, hydrophobic side chains, and the phenol group [35]. In a recently published study, Khallouki et al. found that ATs are agonists for both subtypes of ERs and that they have an effect on estrogen-mediated transcriptional regulation of synthetic and endogenous genes [30]. In our opinion, their conclusion that ATs are a kind of phytoestrogen [30] and their transcriptional ER modulation must be taken into account in pathophysiological pathways including these of UFs is correct.

The above example is not the first observation of estrogen-like properties of AT. Peralta et al. reported that vitamin E increases the expression of estrogenic markers in breast biopsies [65]. These authors found that the biomarkers of estrogen-stimulation were significantly higher in breast biopsies of women who were supplementing vitamin E while being on tamoxifen therapy. Their findings were concurrent with decreased tamoxifen serum levels in same patients [65]. These data suggest that the intake of AT during tamoxifen therapy could impair the clinical outcomes of patients treated with this medicine [66]. Vitamin E might also have some use in the treatment of menopause symptoms [26]. Barton et al. published a randomized clinical trial in which vitamin E was shown to reduce vasomotor symptoms in breast cancer survivors [37]. In more recent publications regarding the potential effect of vitamin E on the female reproductive system, subsequent dependencies have been demonstrated. According to Hashemi et al., supplementation of vitamin E in patients with implantation failure had beneficial effects on the endometrial thickness [36]. Researchers concluded that antioxidant properties of vitamin E are the main influence on stability and thickness [67], in combination with its anticoagulant effect [36, 68, 69]. According to Cicek et al., who studied the effect of vitamin E on controlled ovarian stimulation in women with unexplained infertility, the anticoagulant activity of vitamin E may increase blood supply to the follicles and the proliferating granulosa cells and increase the estrogen production [69]. These authors concluded also that anticoagulant activity of vitamin E may improve the endometrial thickening via better blood flow [69]. It would be interesting to find out whether these processes also occur in UFs. This has not been studied yet, but these results could be also explained by estrogen-like properties of vitamin E. Bafor et al., in a very interesting study about the effects of AT on the concentrations of reproductive hormones in mice, found that treatment with AT significantly increased the plasma levels of luteinizing hormone (LH), estradiol, and progesterone [70]. They also found that AT affected not only the hypophysis alone (effect on LH), but also the ovaries (effect on estrogen and progesterone) [70].

In light of the above-mentioned data, it seems reasonable to conclude that AT and its transcriptional ER modulation must be taken into account to better understand the pathophysiology of UFs. Many patients with clinically symptomatic UFs are known to take antioxidant dietary supplements, which we believe may have a negative impact on the overall clinical outcome. In our opinion and in the opinion of experts, it is necessary firmly address the fact that consumption of dietary supplements has become fashionable (including AT), because it may produce the opposite effect to the one intended, as, for example, is observed in oncology [50, 64, 66].

## 6. Conclusions

In our study, we confirmed that Caucasian women without UFs have lower AT serum concentrations. Higher AT serum concentrations might be an important risk factor for the development of UFs in Caucasian women. Further studies with larger sample size are necessary to confirm these findings.

## Figures and Tables

**Table 1:** Baseline characteristics of studied subjects.

	Control group (n = 62)		UF-positive group (n = 100)	
	Mean ± SD	Median (IQR)	Mean ± SD	Median (IQR)
Age (y)	38.81 ± 7.74	40.00 (32.0; 46.0)	40.20 ±6.59	40.00 (36; 44.5)
Weight (kg)	66.50 ± 12.33	65.00 (58.0; 74.0)	72.06 ±14.44	70.00 *∗* (60.5; 80.0)
Height (cm)	167.58 ± 5.14	167.00 (164.0; 170.0)	166.68 ±5.67	167.00 (162.0; 170.0)
BMI (kg/m^2^)	19.85 ± 3.68	22.31 (20.5; 26.7)	21.61 ±4.22	24.91 *∗∗* (22.0; 29.5)
*α*-tocopherol (*μ*g/ml)	7.83 ± 3.13	7.42 (5.6; 9.1)	11.66 ±4.97	10.56*∗∗∗* (8.5; 13.5)

Significant differences between control and UF group (Mann-Whitney U-test). *∗p *= 0.013; *∗∗p *= 0.005; *∗∗∗p *< 0.001 (n: number of subjects included in each group; y: years; cm: centimeters; kg: kilograms; *μ*g: micrograms; ml: milliliters; SD: standard deviation; IQR: interquartile range; BMI: body mass index; UF: uterine fibroid).

**Table 2:** Association between studied risk factors and uterine fibroids occurrence.

	Control group (n=62 )	UF group (n=100)	Crude			Adjusted for age		
			OR	95% CI	*p*	OR	95% CI	*p*
UF in family, n (%)	9 (14.5%)	42 (42%)	4.26	1.88-9.65	**<0.001**	4.30	1.89-9.76	**<0.001**

Obesity (BMI ≥30kg/m^2^), n (%)	4 (6.5%)	21 (21%)	3.85	1.24-11.93	**0.018**	3.64	1.17-11.36	**0.025**

Delivery (one or more), n (%)	42 (68%)	51 (51%)	0.50	0.25-0.96	**0.037**	0.26	0.11-0.61	**0.002**

*α*-tocopherol, n (%)								
<7.26 *μ*g/ml	29 (48%)	12 (12%)	1.0			1.0		
7.26-9.09 *μ*g/ml	18 (29%)	22 (22%)	2.95	1.16-7.49	**0.021**	2.91	1.11-7.63	**0.027**
9.10-11.92 *μ*g/ml	10 (16%)	31 (31%)	7.49	2.77-20.27	**<0.001**	7.25	2.56-20.51	**<0.001**
>11.92 *μ*g/ml	5 (8%)	35 (35%)	16.92	5.24-54.53	**<0.001**	18.05	4.81-67.79	**<0.001**

Crude and age-adjusted logistic regression analysis. Quartiles of *α*-tocopherol measurements were used in statistical analyses (n: number of subjects included in each group; m: meters; kg: kilograms; *μ*g: micrograms; ml: milliliters; BMI: body mass index; UF: uterine fibroid; OR: odds ratio; and CI: confidence interval).

## Data Availability

The data used to support the findings of this study are available from the corresponding author upon request.
